# Supplementary material for: Characterization of Cell-Bound CA125 on Immune Cell Subtypes of Ovarian Cancer Patients Using a Novel Imaging Platform
Source: Cancers (Basel). 2021 Apr 25;13(9):2072. doi: 10.3390/cancers13092072 (PMC8123299; doi:10.3390/cancers13092072)
Supplement: Supplementary file 1 [file cancers-13-02072-s001.zip › cancers-1132835-supplementary.pdf]

Supplementary Table 1. Percentage of cells that have low, medium, and high nanoparticle binding in serous invasive ovarian cancer patients. Cutoff thresholds of 5 PNPs/cell and 10 PNPs/cell were used to determine medium and high binding.

Cell Type	Low (<5 PNPs/cell)	Medium (5≤PNPs/cell <10)	High (≥ 10 PNPs/cell)
**B-Cell**	40.66	7.59	51.73
**NK-Cell**	85.19	5.21	9.59
**T-Cell**	82.05	5.59	12.35
**Monocyte**	9.82	3.96	86.21
**NK-T-Cell**	71.15	4.83	24.00
